# Exercise-induced mitochondrial remodeling in energy-demanding organs during aging

**DOI:** 10.1016/j.isci.2026.116791

**Published:** 2026-07-15

**Authors:** Zhuoyang Zhou, Jianhong Gao, Minghui Wang, Hu Zhang

**Affiliations:** 1 College of Physical Education and Health, Anhui University of Chinese Medicine, Hefei 230012, China; 2 Graduate School of Anhui University of Chinese Medicine, Hefei 230012, China; 3 College of Sports Medicine, Wuhan Sports University, Wuhan 430079, China; 4 Institute of Acupuncture and Meridians, Anhui University of Chinese Medicine, Hefei 230012, China

## Abstract

Age-associated organ dysfunction markedly impairs quality of life and increases mortality in older adults. Aging frequently results in compromised mitochondrial function in organs with high energy demands, such as skeletal muscle, the brain, heart, kidneys, and liver. This impairment leads to excessive production of reactive oxygen species, increased inflammation, energy deficits, and aberrant cellular signaling, collectively fostering cellular senescence, and chronic diseases. Empirical research has demonstrated that regular physical exercise preserves mitochondrial integrity. This review summarizes common and specific responses to exercise in mitochondrial regulation across various organs and provides a comprehensive cross-organ analysis. The objective was to elucidate the molecular mechanisms through which exercise confers anti-aging effects and mitigates degenerative functional decline by restoring mitochondrial homeostasis. This review provides a theoretical foundation for developing targeted anti-aging interventions and for attenuating aging in multiple organs through lifestyle modifications.

## Teaser

Human metabolism; molecular biology; developmental biology

## Introduction

Since ancient times, humans have sought methods to delay aging and promote longevity. However, aging is an inevitable process that leads to a progressive decline in the functions of various tissues and organs, including skeletal muscle, liver, brain, heart, and kidneys. This deterioration contributes to multiple chronic diseases, such as aging-related muscle atrophy,^1^ hepatic dysfunction,^2^ neurodegenerative diseases,^3^ heart failure,^4^ and chronic kidney disease (CKD),^5^ all of which represent major causes of mortality among the older adults. With the global population aging rapidly, disorders associated with the degeneration of these tissues and organs are becoming increasingly prevalent and pose a serious threat to public health.^1^^,^^6^^,^^7^^,^^8^^,^^9^ Exercise as a means to combat disease, delay aging, and promote health has been advocated by civilizations throughout history. From early empirical understanding to modern scientific validation, numerous studies have demonstrated that exercise enhances tissue and organ function through the regulation of diverse molecular mechanisms and can partially prevent chronic diseases. Mitochondria, the primary source of energy in eukaryotic cells, are abundant in tissues and organs with high metabolic activity, supporting energy-demanding physiological processes. They participate in essential biological processes, including cellular metabolism, energy production, proliferation, and apoptosis. At rest, the specific metabolic rates (*K*i, kcal·kg^−1^ d^−1^) of the liver, brain, heart, kidney, skeletal muscle, adipose tissue, and other organs are approximately 200, 240, 440, 440, 13, 4.5, and 12, respectively.^10^ These organs rely heavily on mitochondrial function and are therefore particularly susceptible to aging. The decline in mitochondrial quantity, function, and quality is considered a key factor driving both aging and chronic diseases.^11^^,^^12^^,^^13^ Nevertheless, some studies have reported that mitochondrial characteristics are more strongly influenced by physical activity than by chronological age.^14^^,^^15^ Although exercise is widely recognized to improve mitochondrial quality to promote healthy aging, most current research focuses on single-tissue or single-organ aging patterns. Consequently, a systematic integrated framework is still lacking to clarify both conserved and organ-specific mechanisms underlying exercise-mediated mitochondrial regulation across multiple high-energy-demanding organs. To overcome these limitations, in this article we considered the framework of systemic high-energy-consuming tissues and organs (skeletal muscle, liver, brain, kidney, and heart) in summarizing the molecular mechanisms through which exercise adaptation-mediated modulation of mitochondrial quality delays aging. The rationale is to provide valuable insights for preventing and treating chronic diseases secondary to the functional decline of energy-demanding tissues and also offer a theoretical foundation for identifying potential targets for exogenous interventions aimed at modulating mitochondrial quality.

## Introduction to mitochondria

**Figure 1:**
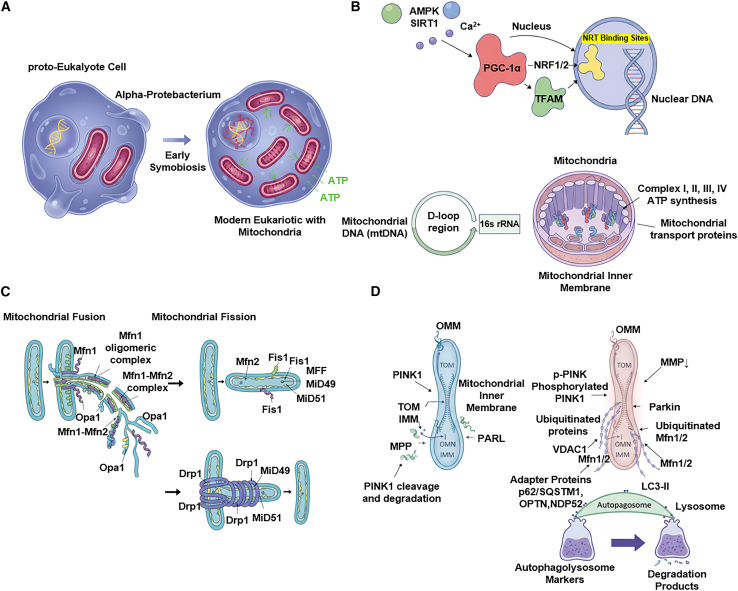
Mechanisms associated with mitochondrial origin, quality control, and function (A) Origin of eukaryotic mitochondria. (B–D) Molecular mechanisms underpinning mitochondrial regeneration, fusion, fission, and mitophagy.

Mitochondria originated from ancient *α*-proteobacteria^16^ that were engulfed, but not digested, by primitive eukaryotes, forming a mutually beneficial symbiotic relationship (Figure 1A). These endosymbionts supplied adenosine triphosphate (ATP) to the eukaryotic host,^17^ providing the energetic foundation for the emergence of functionally complex eukaryotic organisms. The maintenance of normal mitochondrial function fundamentally relies on mitochondrial quality, which is primarily ensured through mechanisms including mitochondrial biosynthesis, mitochondrial dynamics, mitophagy, and mitochondrial unfolded protein responses (UPRmt).

### Mitochondrial biogenesis

Mitochondrial biosynthesis is primarily mediated by the activation of nuclear transcription coactivator peroxisome proliferator-activated receptor gamma coactivator 1α (PGC-1α), which activates genes encoding respiratory chain complex subunits and mitochondrial transport proteins by nuclear respiratory factors 1 and 2 (NRF1/2). This, in turn, regulates mitochondrial transcription factor A (TFAM), driving replication and transcription of mtDNA, thereby promoting mitochondrial biosynthesis (Figure 1B). During this process, newly formed mitochondria undergo continuous morphological and functional remodeling, including fusion, fission, and autophagy, in response to external energy demands and internal optimization cues.

### Mitochondrial fusion and fission

Mitochondrial fusion is mediated primarily by three guanosine triphosphatases (GTPases), mitofusin 1 (Mfn1), mitofusin 2 (Mfn2), and optic atrophy protein 1 (Opa1) (Figure 1C). Mfn1 and Mfn2 form homotypic and heterotypic oligomeric complexes (Mfn1-Mfn1, Mfn1-Mfn2, Mfn2-Mfn2) that mediate outer membrane fusion,^18^ whereas Opa1 interacts with itself and with cardiolipin to mediate inner membrane fusion.^19^^,^^20^ Fusion can generate elongated mitochondrial networks through end-to-end joining of separate mitochondria or form ring structures by the fusion of opposite ends of the same mitochondrion. Conversely, mitochondrial fission primarily depends on the recruitment of the GTPase dynamin-related protein 1 (Drp1). Drp1 assembles into a helical structure on the mitochondrial surface, constricting and severing the membrane to complete fission. Fission 1 (Fis1), mitochondrial fission factor (MFF), and mitochondrial dynamics proteins of 49/51 kDa (MiD49/51) facilitate Drp1 recruitment and act as its receptors, thereby determining the efficiency and localization of mitochondrial fission.

### Mitophagy

Several signaling pathways mediate mitophagy, including the classical PTEN-induced kinase 1 (PINK1)/parkin RBR E3 ubiquitin protein ligase (parkin), BCL2/adenovirus E1B 19 kDa protein-interacting protein 3 (BNIP3)/Nip3-like protein X (NIX), and FUN14 domain-containing protein 1 (FUNDC1) pathways.^21^ When mitochondrial membrane potential decreases due to damage, PINK1 import is blocked, which leads to accumulation on the outer membrane, where it phosphorylates parkin (E3 ubiquitin ligase) and ubiquitin (Ub), thereby recruiting parkin to damaged mitochondria. This indirectly recruits LC3-II to encapsulate the mitochondria, where it fuses with lysosomes for degradation.^22^ Upon mitochondrial damage, BNIP3/NIX recruits autophagosomes by binding to LC3, thereby mediating the clearance of dysfunctional mitochondria (Figure 1D). Similarly, FUNDC1-mediated mitophagy is activated under hypoxic conditions. FUNDC1 interacts directly with LC3 in the cytoplasm through its N-terminal YKL sequence to initiate selective mitochondrial degradation, whereas its C-terminus contributes to protein stability within the intermembrane space.^23^

### Mitochondrial unfolded protein response

UPRmt is a highly conserved cellular stress mechanism that plays a crucial role in maintaining protein homeostasis within mitochondria.^24^ In response to various mitochondrial stressors, such as oxidative stress, protein misfolding, mitochondrial DNA damage, or respiratory chain dysfunction, the accumulation of unfolded or misfolded proteins in the mitochondrial matrix triggers the activation of UPRmt. This response involves a mitochondria-to-nucleus retrograde signaling pathway, which leads to the upregulation of a set of nuclear-encoded genes. These genes include those encoding mitochondrial molecular chaperones, such as heat shock protein 60 (HSP60) and (HSP10), and proteases, such as lon protease 1 (LONP1) and caseinolytic protease mitochondrial (CLPP). The upregulation of these genes enhances the protein folding capacity and facilitates the removal of damaged proteins, thereby contributing to the restoration of mitochondrial function.^25^^,^^26^^,^^27^

## Mitochondrial function

Mitochondria are double-membrane-bound organelles commonly found in eukaryotic cells. They are primarily responsible for supplying cellular ATP by utilizing pyruvate, acetyl-coenzyme A, fatty acids, and amino acids as substrates. Mitochondria exhibit a high degree of flexibility in their morphology, structure, quantity, and quality. Structurally, mitochondria consist of the outer mitochondrial membrane, inner mitochondrial membrane, and mitochondrial matrix, each containing numerous functional proteins and enzymes essential for normal cellular metabolism.

The outer mitochondrial membrane is composed of a phospholipid bilayer characterized by high porin content, and low cholesterol levels, which cf. plasticity, structural integrity, and selective permeability. Functioning as a physical barrier, the outer membrane maintains mitochondrial integrity and separates the organelle from the cytoplasmic environment. However, it allows passive diffusion of small molecules (<5 kDa), such as ATP, adenosine diphosphate (ADP), pyruvate, and various metabolites, through porin channels, facilitating the exchange of materials and energy between the mitochondria.^28^ Damage to the outer mitochondrial membrane disrupts its structural integrity and promotes the release of cytochrome c (Cyt-C) or mtDNA, triggering apoptotic and inflammatory responses that compromise cellular function and survival.

The inner mitochondrial membrane also consists of a phospholipid bilayer, similar to the outer membrane; however, its area is substantially larger than that of the outer membrane owing to the presence of mitochondrial cristae. The expansion of this membrane provides a structural foundation for the bioenergetic function of mitochondria by accommodating a high density of electron transport chain (ETC) complexes and ATP synthase binding sites required for OXPHOS, energy conversion, and ion regulation. The ETC comprises complexes I (NADH:ubiquinone oxidoreductase), II (succinate:ubiquinone oxidoreductase), III (Cyt-C oxidoreductase), and IV (Cyt-C oxidase). NADH and FADH_2_ donate electrons to ubiquinone (CoQ) via complexes I (which pumps 4 H^+^) and II, respectively. Electrons are subsequently transferred through complex III (Q cycle, pumping 4 H^+^) and Cyt *c* to complex IV (pumping 2 H^+^), where O_2_ is ultimately reduced to H_2_O. The energy released during electron transport drives proton translocation from the matrix to the membrane gap, forming a proton gradient (ΔμH^+^, containing ΔΨ and ΔpH). ATP synthase uses the kinetic energy of H^+^ backflow (1 ATP molecule is synthesized for every 3 H^+^) to catalyze ADP + Pi → ATP through conformational changes in the F_1_ unit (combined with an allosteric mechanism). Each molecule of NADH and flavin adenine dinucleotide (FADH_2_) supports the synthesis of approximately 2.5 and 1.5 ATP molecules, respectively. The coupling efficiency of OXPHOS is modulated by ADP availability, uncoupling proteins (such as uncoupling protein 1 [UCP1]), and reactive oxygen species (ROS).^29^ Moreover, the formation of supercomplexes (e.g., I + III_2_ + IV) enhances electron transfer efficiency and minimizes ROS leakage during oxidative phosphorylation. The mitochondrial matrix is a viscous compartment enclosed by the inner membrane that contains mtDNA, enzymes, and metabolites. It serves as a central site for key biochemical processes, including the tricarboxylic acid (TCA) cycle, mitochondrial protein synthesis, and ROS scavenging.

## Biological processes associated with mitochondria

While providing ATP to cells, mitochondria also regulate multiple molecular biological processes, including apoptosis, neuroinflammation, ROS levels, and calcium ion homeostasis. Maintaining a balance among these processes is essential for preserving cellular, tissue, and organ function, as well as for influencing aging and disease progression.

### Apoptosis

**Figure 2:**
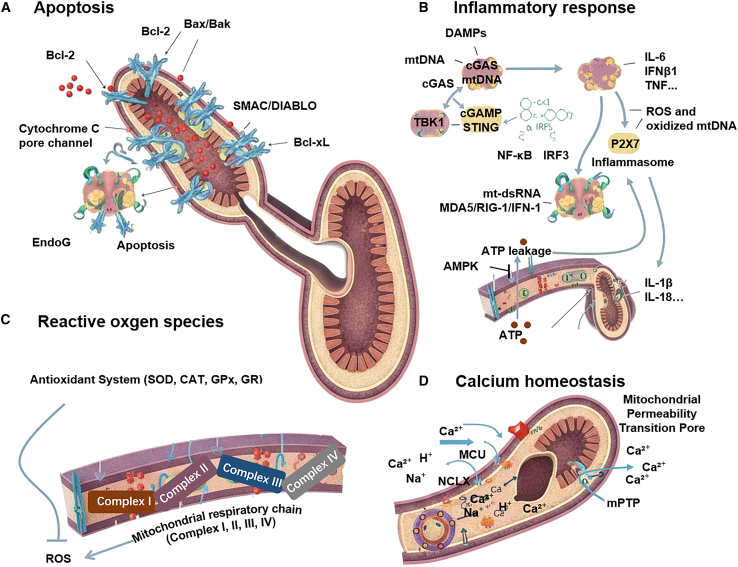
Primary physiological functions of mitochondria The central role of mitochondria in maintaining cellular homeostasis and aging is primarily reflected in four phenomena: (A) apoptosis: activation of the caspase cascade through the release of factors, such as Cyt-C, SMAC/DIABLO, and AIF. (B) Inflammatory response: damaged mitochondria release DAMPs, such as mtDNA, ATP, and ROS, which can activate inflammasomes and induce the release of inflammatory factors. (C) Oxidative stress: imbalance in the function of the ETC leads to increased ROS generation, which damages proteins, lipids, and DNA, accelerating aging and disease progression. (D) Calcium homeostasis: regulation of intracellular calcium signaling through calcium transport and mPTP channels, which can trigger cell damage when overloaded.

Mitochondria serve as the primary induction and response platform for intrinsic apoptosis. When mitochondria are damaged or stimulated by apoptotic signals, the pro-apoptotic proteins Bax and Bak become activated and form oligomeric channels on the outer membrane, thereby increasing mitochondrial outer membrane permeability (MOMP). This process facilitates the release of apoptotic factors, such as Cyt-C (Figure 2A), second mitochondria-derived activator of caspases/direct inhibitor of apoptosis protein (IAP)-binding protein with low pI (SMAC/DIABLO), apoptosis-inducing factor (AIF), endonuclease G (EndoG), and OMI, into the cytoplasm, initiating the apoptotic cascade. Although anti-apoptotic proteins, such as B cell lymphoma 2 (Bcl-2) and B cell lymphoma-extra-large (Bcl-xL), inhibit mitochondrial outer membrane disruption by binding to Bcl-2-associated X protein (Bax) and Bak proteins and participate in apoptosis inhibition via MCL-1, Bcl-2-like 2 (BCL-W), Bcl-2-related protein expressed in fetal liver-1 (BFL-1), and Bcl-2-like 10 (BCL-B), cells ultimately undergo apoptosis when pro-apoptotic signals dominate.^30^ Cyt-C forms a heptameric complex (apoptosome) with apoptotic protease activating factor 1 and cysteine-aspartic protease 9 (caspase-9), activating caspase-9 and subsequently inducing downstream cysteine-aspartic protease 3/7 (caspase-3/7) to execute apoptosis. SMAC/DIABLO promotes apoptosis by forming a homodimer with a specific NH2-terminal motif composed of four amino acids (Ala-Val-Pro-Ile) and binding to antagonistic IAPs to inhibit caspases^31^^,^^32^ and by promoting the release of caspase3/9 by interacting with the Baculoviral IAP repeat-containing protein 2 (BIR2) and baculoviral IAP repeat-containing protein 3 (BIR3) domains of X-linked inhibitor of apoptosis proteins (xIAPs).^33^ Under MOMP-disrupting conditions, AIF is released from the inner mitochondrial membrane and converted into its soluble mature form by calcium-activated neutral proteases or caspases, which cleave the peptide bond between Leu101 and Gly102 in its transmembrane anchor domain. Mature AIF translocates to the nucleus, where it recruits and regulates nonspecific endonucleases to cleave DNA into 2–50 kb fragments, ultimately resulting in chromatin condensation. Similarly, the mature 27 kDa EndoG translocates directly to the nucleus to cleave DNA and induce apoptosis.^34^ Therefore, structural damage to mitochondria can lead to the release of multiple pro-apoptotic molecules, triggering apoptosis through distinct pathways.

### Inflammatory response

Mitochondria are not only central organelles for energy production but also play key roles in activating inflammasomes (such as NOD-, LRR-, and pyrin domain-containing protein 3 [NLRP3]) and immune signaling pathways by releasing damage-associated molecular patterns (DAMPs) to initiate inflammatory responses. Structural damage to mitochondria leads to the release of various DAMPs into the cytoplasm or extracellular environment, promoting inflammation. Typical DAMPs include ATP, ROS, double-stranded RNA (dsRNA), mitochondrial RNA (mtRNA), and mtDNA^35^). When *mtDNA* leaks into the cytosol through mitochondrial channels, it is recognized by cyclic guanosine monophosphate-adenosine monophosphate synthase (cGAS) to form a cGAS-mtDNA complex (Figure 2B), which catalyzes the production of cyclic GMP-AMP (cGAMP). The cGAMP-STING complex then binds to TANK-binding kinase 1 (TBK1) to activate nuclear factor kappa-light-chain-enhancer of activated B cells (NF-κB) and interferon regulatory factor 3 (IRF3), promoting the transcription of inflammatory and type I interferon genes and inducing cytokines, such as interferon-β1 (IFNβ1), interleukin-6 (IL-6), and tumor necrosis factor (TNF).^36^ In addition, oxidized *mtDNA* and ROS can sustain ATP levels directly or through creatine phosphate, activate the purinergic receptor P2X and ligand-gated ion channel 7 (P2X7), and initiate inflammasome assembly.^37^ Conversely, activation of AMP-activated protein kinase (AMPK) reduces extracellular ATP leakage and inhibits NLRP3 assembly, thereby decreasing the release of inflammatory factors, such as interleukin-1 beta (IL-1β) and interleukin-18 (IL-18).^38^ The bidirectional regulatory role of ROS in NLRP3 activation appears to be closely linked to intracellular ATP levels; upon ATP depletion, ROS can activate AMPK-mediated mitophagy to suppress inflammasome activation. Additionally, destruction of mitochondrial structure allows mitochondrial double-stranded RNA (mt-dsRNA) to leak into the cytosol, where it induces inflammatory storms via the melanoma differentiation-associated protein 5 (MDA5)/retinoic acid-inducible gene 1 (RIG-1)/type I interferon (IFN-1) pathway, activation of protein kinase R, and Toll-like receptor 3/NF-κB.^39^^,^^40^^,^^41^ The transfer of mitochondrial inner membrane cardiolipin to the outer membrane or its oxidation activates NLRP3, TLR4, and Cyt-C, promoting the release of inflammatory factors, such as IL-1β and TNF-alpha (TNF-α).^42^ Collectively, these results demonstrate that mitochondrial structural disruption and the release of related components play central roles in the initiation and amplification of inflammatory responses.

#### Reactive oxygen species

ROS are primarily generated by the mitochondrial respiratory chain, where they participate in processes, such as the cell cycle, signal transduction, and apoptosis, constituting an important component of mitochondrial function. Mitochondria transfer electrons through the inner membrane respiratory chain complexes (I–IV) to perform OXPHOS, thereby generating energy (ATP) and ROS while reducing oxygen to water. The main sources include direct electron leakage from flavin mononucleotide (FMN) or iron-sulfur clusters reacting with oxygen to form O_2_^−^; additionally, during the “Q cycle,” the semiquinone free radical intermediate of coenzyme Q (ubiquinone) leaks electrons and reacts with oxygen to generate O_2_^−^; and catalytic reactions of monoamine oxidase (MAO) and dihydroorotate dehydrogenase (DHODH) on the inner mitochondrial membrane.^43^ Under typical physiological conditions, approximately 1%–2% of the electrons that escape from the ETC interact with molecular oxygen to generate superoxide anions (·O_2_^−^), which constitute the primary source of mitochondrial ROS.^44^ When the oxidative and antioxidant systems are in balance, mitochondrial ROS can be efficiently neutralized and regulated. However, when mitochondria are damaged and the antioxidant defense system, comprising superoxide dismutase (SOD), catalase (CAT), glutathione peroxidase (GPx), and glutathione reductase (GR), is impaired, ROS production increases while clearance is inhibited (Figure 2C). This imbalance leads to irreversible damage to biological macromolecules, such as lipids, DNA, and proteins, ultimately causing cell death and inducing or exacerbating the occurrence of diseases.^45^ Mitochondria produce appropriate amounts of ROS to maintain cellular homeostasis, whereas excessive generation induces oxidative stress, damages cells, and promotes diseases. With ongoing research being continuously enriched, relevant physiological effects have been gradually clarified.^46^ These findings suggest that maintaining ROS homeostasis in specific tissues and organs may play a crucial role in sustaining health and preventing or treating disease.

#### Calcium homeostasis regulation

Calcium ions participate in numerous intracellular physiological processes, including signal transduction, muscle contraction, and neuronal transmission, and represent essential ionic components within cells. As the central organelles responsible for calcium regulation, mitochondria maintain the dynamic balance of intracellular calcium ions (Ca^2+^) through sophisticated uptake and release mechanisms and via coordinated interactions with other organelles.^47^ The primary mechanism of mitochondrial calcium uptake involves the mitochondrial calcium uniporter (MCU) complex (comprising MCU and its regulatory subunits MICU1, MICU2, MICU3, and essential MCU regulator, EMRE) and is influenced by factors, such as cytoplasmic Ca^2+^ concentration and membrane potential (Figure 2D). Upon stimulation, when intracellular calcium levels rise sharply, the MCU complex is rapidly activated to absorb Ca^2+^, thereby reducing cytoplasmic calcium concentration and exerting buffering and protective effects. Mitochondria not only take up Ca^2+^ but also release them to prevent calcium overload that can impair mitochondrial function. This release is mainly facilitated through the mitochondrial sodium-calcium exchanger, mitochondrial permeability transition pore (mPTP), and rapid mitochondrial calcium release channel (RMC).^48^ Under normal physiological conditions, the Na^+^/Ca^2+^ exchanger (NCLX) located in the mitochondrial inner membrane effectively lowers mitochondrial calcium levels and prevents damage by exchanging sodium or hydrogen ions with calcium ions (3Na^+^:1Ca^2+^ or 2H^+^:1Ca^2+^).^49^^,^^50^ However, under stress conditions, the opening of the mPTP results in the massive release of Ca^2+^ into the cytoplasm, accompanied by severe structural and functional damage to mitochondria. Furthermore, mitochondria coordinate with other organelles to regulate calcium levels, primarily through the formation of “contact sites” with the endoplasmic reticulum, the largest intracellular calcium reservoir. After the endoplasmic reticulum releases Ca^2+^ via RyR or IP3R channels, it is promptly taken up by the mitochondrial inner membrane MCU at these contact sites, preventing its diffusion throughout the cytoplasm and causing an increase in calcium ion concentration to disrupt the homeostasis of the intracellular environment.^51^ These observations indicate that mitochondria participate in cell function through the uptake, storage, and release of Ca^2+^.

## Mechanisms underlying exercise-induced remodeling of mitochondrial quality

**Figure 3:**
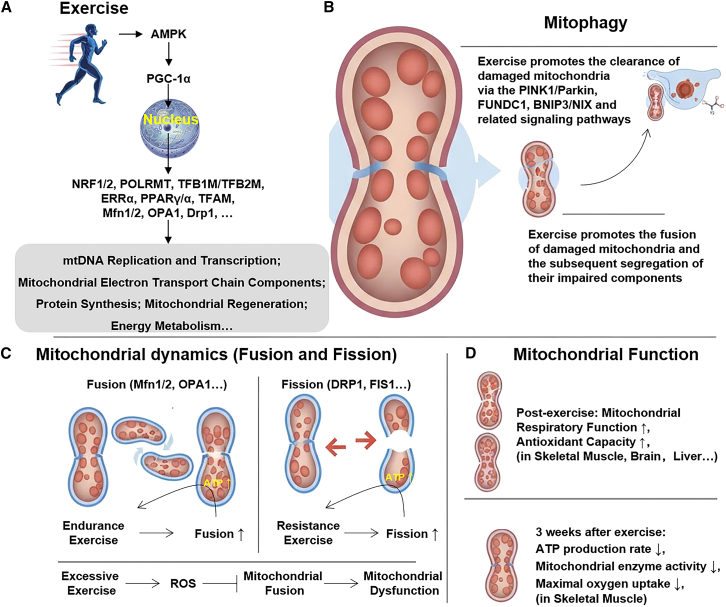
Mechanisms underlying exercise-induced mitochondrial remodeling (A and B) Exercise primarily induces mitochondrial regeneration and function-related protein transcription by activating AMPK and accelerates the clearance of abnormal mitochondria by activating proteins responsible for mitophagy. (C) Aerobic exercise primarily promotes mitochondrial fusion, and resistance exercise tends to promote mitochondrial fission to induce a rapid energy supply. However, excessive exercise can mediate mitochondrial damage. (D) Exercise promotes mitochondrial function and antioxidant capacity in multiple tissues; however, after three months of stopping exercise, mitochondrial ATP production, mitochondrial-related enzyme activity, and other functions decline.

Exercise is a physiological activity with high energy demands. During exercise, the mitochondrial energy requirements of certain tissues and organs can increase by nearly 100-fold.^52^ This heightened demand leads to significant consumption of ATP, resulting in an elevated AMP/ATP ratio. Consequently, AMPK is activated, which facilitates the nuclear translocation of the transcriptional coactivator PGC-1α and regulates the expression of key factors, such as NRF1/2, mitochondrial RNA polymerase (POLRMT), mitochondrial transcription factors B1 and B2 (TFB1M/TFB2M), estrogen-related receptor alpha (ERRα), peroxisome proliferator-activated receptors gamma and alpha (PPARγ/α), TFAM, Mfn1/2, OPA1, and Drp1 (Figure 3A). These molecules are involved in the replication and transcription and mtDNA, synthesis of mitochondrial ETC components and proteins, structural maintenance, and dynamic balance required for mitochondrial biogenesis, as well as the transcriptional regulation of energy metabolism-related genes.^53^ This implies that exercise serves as an important means of regulating mitochondrial quality.

In tissues, such as cardiac and skeletal muscles, exercise enhances Ca^2+^ release from the sarcoplasmic reticulum, activates calcium/calmodulin-dependent protein kinase (CaMK), and phosphorylates OPA1 and Drp1 to facilitate the dynamic regulation of rapid mitochondrial fusion and fission. Moderate levels of ROS generated during exercise promote mitochondrial fission by phosphorylating Drp1, whereas excessive exercise induces large amounts of ROS that inhibit mitochondrial fusion and impair mitochondrial function. Simultaneously, exercise also triggers mitophagy through the activation of signaling pathways, such as PINK1/parkin,^54^ BNIP3/NIX,^55^ and FUNDC1,^56^ which remove damaged, aged, and dysfunctional mitochondria while breaking down mitochondrial components into amino acids, proteins, and other substrates to meet high-intensity energy demands (Figures 3B and 3C). Rapid dynamic remodeling of the mitochondrial network during exercise may, therefore, represent a critical mechanism for the burst production of ATP.

Because long-term endurance exercise primarily depends on aerobic metabolism, mitochondria must maintain a continuous and efficient energy supply, with mitochondrial fusion serving as the principal process that ensures functional stability. During high-intensity exercise, mitochondrial fission increases whereas fusion decreases, allowing mitochondria to rapidly reorganize into multiple cellular nodes to meet the energy demands of short-term, high-energy consumption. During the recovery phase following exercise, mitochondria first undergo fusion to share healthy mtDNA and concentrate damaged components, followed by fission and the activation of mitophagy to remove dysfunctional mitochondria, thereby preventing excessive mitophagic activity. After exercise adaptation, the mitochondrial respiratory function and antioxidant capacity^57^ of tissues and organs, such as skeletal muscle,^58^ brain,^59^ and liver,^60^ exhibit significant enhancement. However, mitochondria are adaptive organelles. When exercise stimulation was stopped for three weeks, the ATP production rate, mitochondrial enzyme activity, and maximal oxygen uptake of skeletal muscle cells significantly decreased (Figure 3D).^61^ These findings suggest that variations in the mode, intensity, and frequency of exercise exert distinct effects on mitochondrial biogenesis, fusion, fission, and autophagy, collectively promoting the renewal and improvement in the quantity, quality, and function of mitochondria.

## Exercise remodels mitochondrial quality and promotes healthy aging

The decline in mitochondrial quality is primarily manifested as reduced mitochondrial respiratory function, regeneration, dynamics, and autophagy, accompanied by increased membrane potential, ROS, and other related substances.^62^ Aging and disease onset in tissues and organs are intricately associated with the decline in mitochondrial quality despite the presence of organ-specific aging patterns. Nevertheless, the regulation of mitochondrial quality serves as a pivotal connection between aging and disease progression.^63^ Therefore, summarizing the key mechanisms through which exercise regulates mitochondrial quality during aging in high-energy-consuming tissues and organs (such as skeletal muscle, liver, brain, heart, and kidneys) may provide insights relevant for delaying organ decline and disease progression.

### Exercise remodels mitochondrial quality to reverse skeletal muscle aging

The skeletal muscle is the largest tissue in the human body, accounting for approximately 40% of body weight. It is essential for maintaining daily activities, metabolism, body temperature, endocrine regulation, and material and energy storage. Skeletal muscle aging is mainly characterized by reduced muscle mass and strength, poor endurance, weakened recovery capacity, and increased risk of diseases, such as sarcopenia and diabetes.^64^ Therefore, delaying skeletal muscle aging can help reduce muscle loss, falls, fractures, and functional decline, thereby promoting healthy aging. Muscle aging is primarily associated with an imbalance between protein synthesis and degradation, reduced mitochondrial content, abnormal autophagy, chronic inflammation, impaired muscle satellite cell function, and hormonal changes.^65^^,^^66^^,^^67^^,^^68^^,^^69^ The accumulation of dysfunctional mitochondria triggers signaling cascades involving ROS, inflammation, and apoptosis, ultimately leading to a marked reduction in skeletal muscle mass.^70^ Mitochondria are key organelles that sustain the high energy demands of skeletal muscle. Exercise plays a critical role in reversing the decline in skeletal muscle mass and function by enhancing mitochondrial quality and has broad potential as an economical and universally applicable intervention.

**Figure 4:**
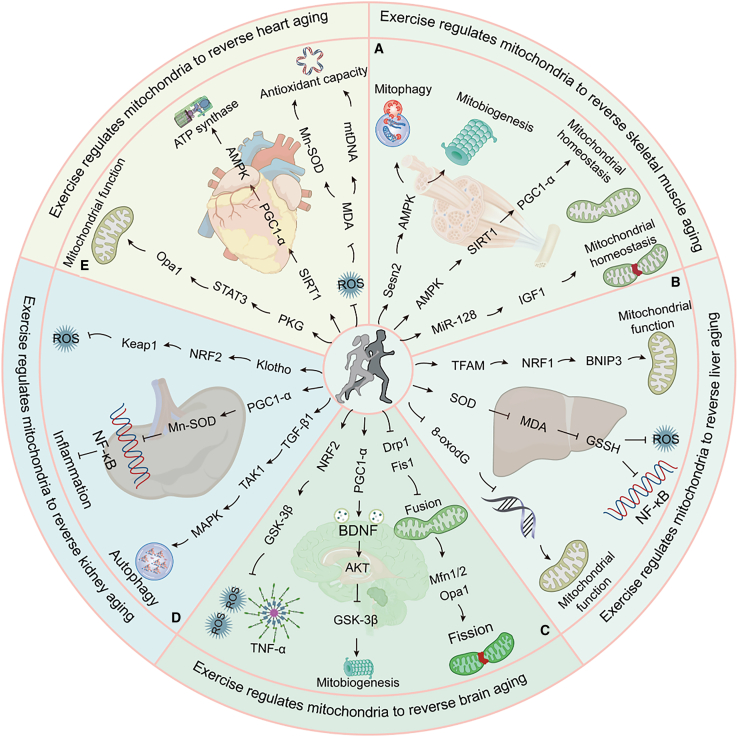
Molecular mechanisms underlying the mitigation of multi-organ aging by exercise via remodeling of mitochondrial quality (A) Exercise mitigates skeletal muscle aging by activating mitochondrial regeneration, mitochondrial autophagy, and maintaining mitochondrial homeostasis via the Sesn2/AMPK, AMPK/SIRT1/PGC-1α, and miR-128/IGF-1 signaling pathways, respectively. (B) Exercise slows liver aging by improving mitochondrial function and inhibiting ROS and NF-κB via the TFAM/Nrf1/BNIP3 and SOD/MDA/GSSH pathways and 8-oxodG, respectively. (C) Exercise mitigates brain aging by promoting mitochondrial fusion and upregulation of the PGC-1α/BDNF/AKT/GSK-3 and NRF2/GSK-3β pathways, promoting mitochondrial biogenesis and inhibiting oxidative stress and inflammation. (D) Exercise mitigates renal aging by activating autophagy, inhibiting inflammation, and oxidative stress via the TGF-β1, TAK1/MAPK, PGC-1α/Mn-SOD/NF-κB, and Klotho/NRF2/Keap1 pathways, respectively. (E) Exercise mitigates cardiac aging by activating PKG/STAT3/Opa1, SIRT1/PGC-1α/AMPK, and ROS/MDA/mtDNA pathways, which improve mitochondrial function, increase ATP synthesis, and alleviate oxidative stress.

In animal studies, mitochondrial mass in skeletal muscle begins to decline in middle age, earlier than the decline in muscle mass and strength.^71^ Mice that begin voluntary resistance wheel exercise in middle age exhibit significant improvements in skeletal muscle mitochondrial function and protection against muscle mass loss.^72^ An 8-week exercise intervention in middle-aged zebrafish has been shown to activate the AMPK/SIRT1/PGC-1α pathway in skeletal muscle, inhibit 15-PGDH expression, and consequently optimize mitochondrial quality while reversing skeletal muscle atrophy (Figure 4A).^73^ Sustaining a long-term exercise regimen stabilizes mitochondrial enzyme activity in rat skeletal muscle, preventing age-related decline.^74^ Our previous research further corroborates that lifelong exercise mitigates the degenerative decline in mitochondrial quality in rats by activating the autophagy pathway.^75^ These findings indicate that age-related skeletal muscle aging is a progressive process that requires early intervention. Notably, even after the onset of aging, exercise intervention can exert a beneficial regulatory effect. Nonetheless, whether exercise maintains mitochondrial enzyme activity indirectly by improving overall mitochondrial quality or through direct regulation of enzyme activity remains unclear.

Various exercise modalities positively influence skeletal muscle mitochondria across different aging models. High-intensity interval training (HIIT) activates skeletal muscle AMPK, enhances mitochondrial stem cell activity, increases levels of mitophagy, and elevates the expression of proteins related to mitochondrial biosynthesis.^76^ Aerobic exercise maintains mitochondrial homeostasis via the miR-128/insulin-like growth factor 1 (IGF-1) pathway^77^ and mitigates D-galactose-induced aging changes in skeletal muscle in murine models. Furthermore, even in 28-month-old mice representing late stages of aging, a combination of aerobic and resistance training ameliorates translational abnormalities in key mitochondrial proteins and improves the quality control system of skeletal muscle proteins.^78^ Collectively, these findings suggest that exercise can modulate skeletal muscle mitochondria throughout aging. However, findings from related studies have been inconsistent. For example, aging attenuates the responsiveness of PGC-1α to exercise, limiting the ability of exercise to reverse the decline in mitochondrial biogenesis^79^; in contrast, PGC-1α overexpression in aging models suppresses mitophagy, thereby indirectly mitigating mitochondrial damage.^80^ Therefore, the mechanism of PGC-1α in the quality control of aging skeletal muscle, especially the resistance to exercise, needs further in-depth research. In summary, basic research shows that the decline in mitochondrial quality begins in the middle age, but exercise intervention at different stages shows positive effects in the vast majority of cases.

Population studies have shown that aging is associated with a general decline in myofibrillar and mitochondrial contents in older adults. However, exercise interventions can effectively mitigate age-related alterations in skeletal muscle.^81^ Analyses of skeletal muscle biopsies have further corroborated that the expression, activity, gene transcription levels, and mitochondrial DNA copy number of proteins related to the mitochondrial respiratory chain complex are markedly lower in the elderly than in younger cohorts. This finding indicates a progressive increase in mitochondrial damage during aging.^82^^,^^83^ Long-term, regular exercise has the potential to reverse the reduction in both volume and number of skeletal muscle mitochondria, thereby delaying the degenerative decline in overall mitochondrial function.^84^^,^^85^ In older individuals who consistently engage in lifelong exercise, oxidative metabolism in skeletal muscle is enhanced, and the adaptive response to acute endurance exercise can be maintained over an extended period.^86^ In those with adequate daily physical activity, the overall expression of oxidative phosphorylation (OXPHOS)-related proteins within the skeletal muscle is upregulated, with the most notable increase observed in Cyt-C oxidase (COX) activity.^87^ This increase directly reflects the beneficial effects of exercise on the mitochondrial oxidative phosphorylation function of the skeletal muscle. Furthermore, even among individuals aged 85 and above who maintain a high level of physical activity, mitochondrial respiratory chain function and skeletal muscle reserves remain in a favorable state, thereby significantly reducing the risk of muscle atrophy and weakness.^88^ Aerobic exercise concurrently optimizes mitochondrial function and preserves total skeletal muscle mass through the sestrin 2 (Sesn2)/AMPKα2 signaling pathway.^88^ Additionally, resistance training positively enhances the efficiency of mitochondrial respiratory transport in older adults.^89^ However, several studies have yielded conflicting results, indicating no fundamental differences in mitochondrial ATP production capacity and oxidative metabolism levels between young and elderly individuals. Moreover, indicators of mitochondrial respiration and dynamic remodeling in sedentary individuals appear largely independent of age.^90^^,^^91^ These inconsistencies are often attributed to limited sample size, inadequate screening for chronic diseases among participants, and considerable heterogeneity in baseline activity levels. Further larger-scale clinical trials incorporating more rigorous baseline stratification are warranted to clarify these findings. Notably, mild abnormalities in skeletal muscle oxygen metabolism have been observed in some elderly individuals with sarcopenia, even when engaging in moderate-intensity exercise at rest, which may aid in mitigating acidosis risk.^92^ This phenomenon provides additional evidence that mitochondrial dysfunction is a fundamental cause of skeletal muscle loss and a critical underlying mechanism contributing to adverse physiological responses, such as exercise intolerance, when exercise intensity is increased in the elderly. These findings support the incorporation of real-time physiological monitoring into exercise intervention programs for sarcopenia to minimize the risk of excessive exercise loads exacerbating mitochondrial damage. Although regular training can markedly enhance the mitochondrial function of skeletal muscle in older adults, compared to younger individuals, the elderly experiences a substantially more rapid decline in mitochondrial-related enzyme activity following the cessation of exercise interventions.^93^

Mitochondrial transplantation plays an important role in the prevention and treatment of skeletal muscle degeneration.^94^^,^^95^ Although this technique remains in its early stages, it demonstrates that mitochondria are key organelles targeted for alleviating skeletal muscle loss.^96^ These findings align with results from systematic reviews of current basic and clinical research, indicating that the enhancement of mitochondrial quality constitutes an essential molecular mechanism through which exercise prevents and mitigates skeletal muscle aging, characterized by decline in muscle mass and strength.^97^

Although some studies, especially in humans, have yielded mixed results, which may be attributed to factors, such as the number of confounding factors, degree of aging, number of experimental samples, exercise dosage, and underlying diseases, the vast majority of studies have confirmed that exercise remodeling of mitochondrial quality is an important mechanism for improving skeletal muscle aging.

### Exercise remodels mitochondrial quality and reverses liver aging

As the largest and most metabolically complex organ in the human body, the liver plays crucial roles in metabolism, detoxification, waste elimination, bile synthesis and secretion, and immunity. Aging can result in a 20%–40% reduction in liver volume,^98^ accompanied by decreased hepatic function and increased lipofuscin accumulation. It may also lead to secondary conditions, such as non-alcoholic fatty liver disease (NAFLD), fibrosis, cirrhosis, and even liver cancer.

Utilizing single-cell detection techniques, a previous study observed that the overall liver tissue in elderly individuals exhibits mitochondrial functional decline and elevated oxidative stress levels.^99^ Morphological observations further corroborate the presence of typical alterations in the aging liver, including reduced mitochondrial numbers, increased volume, and significant lipid accumulation.^100^ Specific mutations in mitochondrial DNA, such as those affecting Cyt-C oxidase subunit 3 (*COX3*), can lead to excessive accumulation of ROS, metabolic disturbances, and mitochondrial fragmentation, thereby accelerating liver aging.^101^ Studies in various animal models have consistently demonstrated that aging can increase hepatic ROS levels, reduce mtDNA copy number, and induce protein damage accumulation.^102^^,^^103^ However, even short-term exercise interventions can markedly reverse age-related liver dysfunctions, inhibit lipid accumulation, and suppress the abnormal upregulation of the senescence marker p53-activated fragment 1 (*p21WAF1*)/CDK-interacting protein 1 (*CIP1*).^104^^,^^105^ Both short-term and long-term exercise regimens can enhance mitochondrial respiratory efficiency, promote mitochondrial biogenesis, and regulate hepatic glucose metabolism, thereby modulating related metabolic signaling pathways.^106^^,^^107^ The protective effects of exercise on liver mitochondria may be partially mediated by enhancements in cardiopulmonary function.^108^ Studies involving aged rats demonstrated that various intervention strategies, such as voluntary wheel exercise, treadmill endurance training, and HIIT, can substantially improve liver mitochondrial respiratory activity and overall metabolic function.^109^ However, the relationship between the regulatory effects of exercise on the aging liver and enhanced systemic oxygen uptake capacity, as well as the underlying molecular pathways, necessitate further comprehensive investigation.

Liver senescence is frequently associated with a disruption in oxidative stress homeostasis, a pathological alteration primarily attributed to the excessive release of mitochondrial-derived ROS. Exercise training of appropriate intensity can mitigate the age-associated decline in the activities of NADH-cytochrome c reductase and cytochrome oxidase in the livers of aged mice, thereby inhibiting the progression of age-related oxidative stress.^110^ Notably, a 2-month regimen of aerobic exercise has been demonstrated to reduce the levels of 8-oxodG in both nuclear and mitochondrial DNA within the livers of 21-month-old mice, aligning these levels with those observed in 11-month-old counterparts, thereby providing direct evidence of the protective effect of exercise against mitochondrial DNA oxidative damage.^111^ Furthermore, a 54-week continuous aerobic exercise intervention reportedly enhances the activities of SOD, CAT, and citrate synthase in the livers of middle-aged mice by 10%, 27%, and 58%, respectively, thereby ameliorating mitochondrial dysfunction.^60^ Comparable outcomes have been observed following an 8-week moderate-intensity treadmill intervention in aged rats, resulting in reduced malondialdehyde (MDA) and glutathione disulfide (GSSG) levels.^112^ Regular exercise also increases the glutathione (GSH)/GSSG ratio in the liver tissue and reduces ROS and NF-κB activity in 28-month-old rats at advanced stages of aging (Figure 4B).^113^

The progression of NAFLD is intrinsically linked to aging, establishing a detrimental cycle that exacerbates hepatic aging. Physical exercise can mitigate and manage this form of fatty liver disease by preserving mitochondrial homeostasis.^114^ Experimental studies in animal models have indicated that exercise enhances the transcriptional expression of genes such as *TFAM*, *NRF1*, and *BNIP3* in compromised liver tissues. Consistent with these findings, human studies have corroborated that sustained physical activity contributes to the prevention and management of liver diseases by modulating mitochondrial markers and autophagy levels.^115^ In addition, inter-organ regulatory mechanisms may contribute to the exercise-induced modulation of hepatic mitophagy. Notably, ovariectomy markedly diminishes the effect of exercise-induced activation of mitophagy in mice with NAFLD.^116^ This finding also suggests that the decline in exercise-induced mitochondrial quality regulation in the aging liver may be related to the aging of other tissues and organs, especially hormone-regulating organs such as the ovary during the aging process, which deserves attention and further research. Current evidence indicates that exercise prevents and ameliorates liver aging by modulating mitochondrial quality, primarily through the regulation of mitochondrial function, autophagy, and ROS production.

### Exercise remodels mitochondrial quality to reverse brain aging

Although the brain accounts for only approximately 2% of total body weight, it consumes up to 20% of the body’s daily energy (approximately 240 kcal/kg),^10^^,^^117^ rendering it highly dependent on mitochondrial function. During aging, most individuals experience reduced brain volume, decreased neuronal regeneration capacity, and impaired cognitive function, with reduced mitochondrial quality serving as an important molecular mechanism underlying these changes. In the aging brain, mitochondria exhibit decreased activity of respiratory complexes and OXPHOS levels, alongside elevated oxidative stress, inflammation, and apoptosis. The decline in mitochondrial quality is not only a critical pathogenic factor in many neurodegenerative diseases but also progresses more slowly in the brain than in other tissues.^118^^,^^119^ Notably, exercise interventions exert positive regulatory effects on brain mitochondria and can delay aging-related functional decline to a certain extent.^120^^,^^121^

Animal studies employing various intervention protocols have consistently demonstrated that exercise contributes to cognitive protection by regulating hippocampal mitochondrial homeostasis. Specifically, a 7-week swimming training regimen can optimize mitochondrial biosynthesis, maintain the balance of fusion and fission, and initiate mitophagy in 18-month-old rats, effectively reversing age-related cognitive decline.^122^ Similarly, administering a 5-week resistance training program to 18-month-old mice during postoperative recovery activates the hippocampal PGC-1α/BDNF/Akt/GSK-3β signaling pathway. This activation enhances mitochondrial biosynthesis, restored mitochondrial dynamics disorders, and ultimately improves cognitive function (Figure 4C).^123^ These studies indicate that exercise exerts beneficial effects on cognitive rehabilitation in aging and on the prevention of cognitive decline, closely associated with improvements in mitochondrial quality. Another study using a mouse model of premature aging induced by mtDNA mutations demonstrated that voluntary exercise significantly reversed reductions in mitochondria-related proteins involved in the tricarboxylic acid cycle and coenzyme Q metabolism and reduced the mtDNA mutation load in aged mice.^124^ This further confirms that mitochondrial abnormalities can accelerate or trigger aging phenotypes and that exercise intervention can alleviate these effects to some extent.

In an exogenous aging model induced by D-galactose, aerobic exercise mitigates oxidative stress and neuroinflammation, enhances mitochondrial quality control mechanisms, and delays cognitive decline.^125^ Under this exercise regimen, aerobic exercise downregulates the expression of mitochondrial fission proteins Drp1 and Fis1 while upregulating fusion proteins such as Mfn1, Mfn2, and Opa1. Concurrently, it modulates the redox system, which includes NADPH oxidase, SOD-2, and catalase, thereby inhibiting COX-2- and TNF-α-mediated neuroinflammatory pathways and enhancing cognitive function.^126^ These effects may involve mitochondrial antioxidant regulation mediated by the NRF2/GSK3β signaling pathway.^127^ These findings suggest that exogenous drug-induced aging occurs through impairment of mitochondrial quality and that exercise can effectively counteract cognitive decline by improving mitochondrial function. Notably, in models of age-related cognitive impairment such as Alzheimer’s disease (AD), exercise interventions enhance cognitive function primarily through substantial improvements in mitochondrial quality.^121^^,^^128^^,^^129^^,^^130^

Nonetheless, related studies demonstrate variability in their findings. Evidence indicates that 7 weeks of HIIT in middle-aged mice results in abnormal mitochondrial respiratory chain complexes and disrupted mitophagy.^131^ Comparable mitochondrial damage has been reported in studies examining high-intensity exercise interventions in other tissues.^132^ Research involving aged female rats revealed that aging and prolonged exercise intervention do not markedly alter the content, function, or dynamics of mitochondria in the cerebral cortex and cerebellum. This finding indicates that the physiological characteristics and redox homeostasis of female brain mitochondria possess inherent anti-aging properties, which are modulated by both exercise intensity and intervention duration.^133^ Different exercise regimens exert distinct effects on hippocampal proteins involved in mitochondrial metabolism in rats.^134^ Additionally, exercise-induced lactate accumulation can promote mitochondrial biosynthesis.^135^ Furthermore, even in older female rats with reduced motor function, elevated levels of mitochondrial oxidative phosphorylation proteins were observed in the brain tissue.^128^ Therefore, such differential results should take into account the sex and exercise dosage.

Exercise can improve the quality of brain mitochondria, whether caused by medication or natural aging. However, in exercise interventions for aging, careful control of the dosage and intensity of exercise is important, otherwise, it may exacerbate mitochondrial damage and hinder the improvement of brain function.

### Exercise remodels mitochondria quality and reverses kidney aging

As the principal organs of metabolism and filtration, the kidneys are responsible for urine production and multiple physiological processes essential for maintaining homeostasis. Aging leads to degeneration and marked decline in renal structure and function, mainly manifested by conditions, such as interstitial fibrosis, glomerulosclerosis, tubular atrophy, and arteriosclerosis, thereby greatly increasing the incidence of kidney disease.^136^^,^^137^

Clinical trials investigating exercise interventions in the elderly have demonstrated that parameters associated with glomerular injury, renal tubular dysfunction, renal tubular damage, systemic inflammation, and tubulointerstitial remodeling and fibrosis can be positively modulated in individuals with higher levels of daily physical activity.^138^ An analysis of an adult cohort comprising nearly 200,000 participants further indicated an inverse relationship between daily physical activity levels and the risk of developing CKD. Across low, medium, and high activity gradients, increased activity levels are associated with a reduced probability of disease occurrence.^139^ This finding is corroborated by a secondary analysis of randomized clinical trials involving individuals aged 70–89 years, showing that moderate-intensity exercise can effectively slow the age-related decline in kidney function.^140^ Comprehensive population studies consistently suggest that exercise can retard the aging of the human kidneys, with moderate-intensity interventions demonstrating particularly pronounced benefits.

However, results from basic research are not entirely consistent. A previous study using a rat model of age-related nephropathy found that a prolonged exercise intervention does not reverse disease progression or affect tissue hydrogen peroxide (H_2_O_2_) levels.^141^ Following a controlled intervention involving both forced and voluntary exercise in elderly mice, only voluntary exercise mitigated age-related kidney damage, whereas forced exercise did not demonstrate a protective effect on renal health.^142^ These findings suggest that autonomous exercise modalities are more beneficial than forced or high-intensity exercise regimens in maintaining renal physiological homeostasis. Existing research findings at the molecular regulatory mechanism level suggest that exercise can simultaneously modulate renal aging-related pathological changes through multiple signaling pathways. For instance, the regulation of the Klotho and NRF2/Kelch-like ECH-associated protein 1 (KEAP1) pathways^143^ mitigates oxidative stress, inflammatory infiltration, and apoptosis in aging renal tissues while inhibiting the transforming growth factor β1 (TGF-β1)/TGF-β-activated kinase 1 (TAK1)/MAP kinase kinase 3 (MKK3)/p38 mitogen-activated protein kinase (p38 MAPK) pathway activity and activating autophagy. This phenomenon reduces extracellular matrix accumulation, prevents renal tubular epithelial-to-mesenchymal transition, and ultimately inhibits renal fibrosis progression.^144^ Additionally, exercise relies on the PI3K/Akt/mammalian target of rapamycin (mTOR) pathway to ameliorate pathological changes associated with renal vascular sclerosis in aged mice (Figure 4D).^145^ Further evidence from a 12-week moderate-intensity swimming intervention in 21-month-old rats demonstrated that this exercise regimen can activate PGC-1α, enhance Mn-SOD activity, inhibit NF-κB pathway activation, decrease renal fibrosis and MDA content, and concurrently reduce the expression of renal pro-inflammatory factors, including MCP-1, IL-1β, and IL-6 (Table 1).^146^

**Table 1:** Exercise-mediated regulation of mitochondrial mechanisms in aging high-energy-consuming organs

Study subjects	Disease model	Age	Intervention mode	Intervention dose/Duration	Molecular mechanism(s)	Reference
C57BL/6J mice	sarcopenia/muscle aging	2–20 months	natural aging	N/A	altered mitochondrial dynamics (Mfn1, Mfn2, Opa1, and Drp1); Ca^2+^ dyshomeostasis	Del Campo et al.^71^
C57BL/6J mice	sarcopenia	15 months; 23 months	RWE	34 weeks; resistance increased from 0 to 6 g	increased mitochondrial density and oxidative capacity (CS, NADH-TR); Elevated autophagy (LC3II/I)	White et al.^72^
C57BL/6 mice and C2C12 cells	sarcopenia	6 months; 12 months	aerobic treadmill exercise; Overexpression of Sesn2	8 months; 5 days/week; 60 min/day (75% VO_2_ max)	activation of the Sesn2/AMPKα2 pathway; balanced mitochondrial fusion/fission; improved biogenesis and mitophagy	Liu et al.^88^
C57BL/6 mice and C2C12 cells	sarcopenia	3 months; 24–28 months (young)	exercise (VR); PGC-1α gain/loss of function	lifelong or targeted PGC-1α induction	PGC-1α/ERRα jointly regulated mitochondrial translation machinery; rectification of age-related mitochondrial translation impairment	de Smalen et al.^78^
C57BL/6J mice	sarcopenia/muscle atrophy	2 months; 24 months	*In vivo* PGC-1α DNA transfection (electroporation)	5 days post-transfection	PGC-1α OE downregulated intensified mitophagy (PINK1, Parkin, p62, and LC3II); enhanced antioxidant capacity (SOD2 and CAT)	Yeo et al.^80^
C57BL/6 mice	sarcopenia/exercise damage	3 months; 20 months	strenuous exercise	single bout or chronic (treadmill, high-intensity)	strenuous exercise in old mice induced mitochondrial damage via NF-κB activation and high ROS levels; failed to reverse age-related dysfunction	Lee et al.^132^
Mfn1-mKO mice and C2C12 cells	mitochondrial dynamics disruption	adult mice/cell models	anti-inflammatory treatment; Mfn1/2 or Drp1/Fis1 KD	chronic treatment *in vivo*; acute KD *in vitro*	mtDNA mislocation triggered sterile inflammation via TLR9 (fission) or cGAS (elongation) sensors	Irazoki et al.^147^
Polg mutator (POLG) mice	PMM	9 months (start treatment)	GDF15 neutralizing antibody (mAB2)	12 weeks; once weekly subcutaneous (10 mg/kg)	reversal of transcriptional dysregulation in autophagy/proteasome signaling	Flaherty et al.^148^
SD rats (female)	sarcopenia	8 months; 26 months	HIIT vs. MICT	8 months; 5 days/week; 45 min/day	HIIT activated the AMPK pathway; upregulated the mitoSCs (Supercomplexes) assembly, biogenesis, and mitophagy markers (irisin and SOD2)	Han et al.^76^
Wistar rats and PGC-1α KO mice	sarcopenia/Frailty	rats: 3 and 24 months; Mice: 5–6 months	exercise training; Cold induction; T3 treatment	training: 5 days/wk at 75% VO_2_max; Cold: 4°C; T3: 1.5 μg/100 g body wt	age-associated lack of PGC-1α response to exercise; loss of NRF-1 and cytochrome *c* expression in aged rats	Derbré et al.^79^
LCR rats	aging and exercise intolerance	18 months	mitochondrial transplantation (Intramuscular injection)	7.5 × 10^8^, 3.75 × 10^8^, and 5.0 × 10^8^ mitochondria were injected into the quadriceps femoris, tibialis anterior, and gastrocnemius muscles, respectively	rescued muscle bioenergetics; increased mitochondrial markers (CS and ATP); global and site-specific hypomethylation of TFAM	Arroum et al.^95^
Human (male)	sarcopenia/pre-frailty	20–94 years	habitual physical activity (active vs. sedentary)	long-term lifestyle	attenuation of mitochondrial loss (SDH)	St-Jean-Pelletier et al.^81^
Human (older adults)	muscle aging	59 ± 4 years	full-body RT	10 weeks; 2 sessions/week; 3 sets of 10–12 reps	increased OXPHOS complexes (I–V); Upregulation of fusion (Mfn1, Mfn2, Opa1) and fission (Drp1) proteins	Mesquita et al.^149^
Human (young and old)	muscle aging	18–30 years; ≥65 years	aerobic endurance training	8 weeks; 65% VO_2_peak, 60 min, 3–5 days/week	enhanced antioxidant enzymes (SOD2 and CAT) and SIRT3; Buffering against oxidative damage	Johnson et al.^150^
Human (male)	muscle aging	∼27 years; ∼70 years	lifelong physical exercise	long-term (>30 years)	protected cell ultrastructure for Ca^2+^ handling and ATP production; Reduced autophagy gene expression	Zampieri et al.^84^
Human (male and female)	sarcopenia/aging	∼25 years; ∼73 years	habitual physical activity (high vs. low)	long-term lifestyle	improved mitochondrial oxidative capacity (measured by 31P MRS PCr/ADP recovery times)	Waters et al.^85^
Human (male and female)	metabolic dysfunction/aging	71.3 ± 4 years	lifelong ET vs. acute exercise	20–50 years (lifelong); single bout at 75% VO_2_max	higher mitochondrial enzyme activities (PDH, CS, and SDH) and capillarization; blunted acute mRNA response (PGC-1α and VEGF) in trained subjects	Iversen et al.^86^
Human (male)	muscle aging	22 ± 1 year; 67 ± 1 year	regular endurance exercise (active vs. sedentary)	≥6 h/week for at least 5 years	increased mitochondrial fission (Fis1) and mitophagy (PARKIN) protein localization; Enhanced OXPHOS and fusion protein levels	Balan et al.^87^
Zebrafish (*Danio rerio*)	age-related sarcopenia	21 months	swimming exercise intervention	8 weeks; 5 days/week; daily exercise	activation of AMPK/SIRT1/PGC-1α axis; 15-PGDH downregulation; reduced oxidative stress	Sun et al.^73^
Human (middle-aged)	muscle aging	50 ± 7 years	progressive RT	10 weeks; 3 days/week; 5 sets of 10 reps (65% 1-RM)	increased mitochondrial respiratory capacity correlated with strength gains	McKenna et al.^89^
Zebrafish (*Danio rerio*)	D-Galactose induced sarcopenia	7–8 months	aerobic exercise (swimming)	4 weeks; 5 days/week; 4 h/day (25.6 cm/s)	enhanced mitochondrial respiration and homeostasis; reduced tissue fibrosis and ROS	Chen et al.^77^
Human (sedentary and active)	normal aging/BMI influence	21–88 years	cross-sectional observational	sedentary vs. physically active (3–5 sessions/week)	mitochondrial respiration/dynamics (fusion/fission) were associated with BMI and fitness, not with chronological age alone	Rasmussen et al.^90^
Humans (elderly)	sarcopenia/muscle aging	older adults	habitual physical activity	life-long activity	reduced resting ATP/ADP ratio and increased ATP hydrolysis; impaired aerobic metabolism during exercise without acidosis	Waters et al.^92^
Humans (young and elderly)	sarcopenia/aging	24 ± 3 years; 80 ± 4 years	aerobic exercise training and deconditioning	6 weeks training (4 sessions/week, 35 min); 8 weeks deconditioning	increased citrate synthase (CS) and complex I-IV activity with training; faster decline in enzyme activity during deconditioning in the elderly	Fritzen et al.^93^
C57BL/6 mice	liver aging	3 months; 24 months	CR	30% reduction in food intake for 10weeks +1	aging increased inflammatory cells/cytokines and upregulated biosynthesis/metabolism pathways; CR reversed these changes	Lin et al.^99^
mtNOD conplastic mice	premature liver aging	3–18 months	mtDNA mutation study	N/A	mtDNA mutation in COX subunit 3 leads to persistent ROS production and elongated mitochondrial networks; depressed autophagy	Niemann et al.^101^
C57BL/6J mice	D-Gal-induced liver aging	3 months	aerobic treadmill exercise	2 weeks; 5 days/week; 60 min/day (increasing intensity)	counteracted liver inflammation and metabolic shifts; improved physical performance markers	Pinto et al.^104^
C57BL/6 mice	obesity-related MASLD/Senescence	8 weeks; 18 months	exercise vs. DR	8 weeks exercise (treadmill) vs. 40% DR	exercise reduced hepatic senescence (p21 and p16) and fibrosis	Katsarou et al.^105^
C57BL/6 male mice	aging (genomic/metabolic)	6 months; 22 months	HIIT vs. MICT (endurance)	4 weeks; daily sessions (intermittent vs. continuous)	MICT improved body composition and muscle strength in old mice; HIIT showed less clear benefits; structural adaptations in mitochondria	Bernier et al.^106^
C57BL/6 mice	aging/survival	28–78 weeks old	moderate treadmill exercise	weekly sessions; 10–20 cm/s for 5 min	extended life span (19%); decreased oxidative stress (TBARS); enhanced mitochondrial complexes I, II, and IV activity	Navarro et al.^110^
C57BL/6J mice	liver aging/senescence	3 months; 22–24 months	combined physical exercise (aerobic + resistance)	8 weeks; 5 days/week; treadmill + strength training	reversed the age-related reduction of Bmal1; restored hepatic circadian clock and mitochondrial biogenesis (PGC-1α)	Pinto et al.^115^
Female C57BL/6J mice	MASLD/menopause (ovarian loss)	12–15 weeks	OVX + HFD + acute exercise	4 weeks diet; Single bout of treadmill exercise	estrogen deficiency (via OVX) blunted exercise-induced activation of hepatic mitophagic flux (selective degradation of mitochondria)	Franczak et al.^116^
Fischer 344 rats	liver and cardiac aging	6–30 months	observational/aging	N/A	age-related accumulation of dense bodies and lipofuscin; increased lysosomal enzyme β-glucuronidase	Schmucker and Sachs^100^
Rhesus monkeys	liver aging	0.6–8 years (young); 9–17 years (middle aged); >19 years (old)	observational/aging	N/A	age-correlated increase in mtDNA lesions/damage and lipid peroxidation; reduced antioxidant enzyme activity	Castro et al.^102^
Fischer 344 rats	liver aging	6 months; 27 months	observational/aging	N/A	substantial age-related reduction of mtDNA copy number in skeletal muscle (−23 to 40%) and liver (−50%); tissue-specific effects	Barazzoni et al.^103^
SD rats (female)	aging/SASP/lipolysis	3 months; 24 months	lifelong exercise and detraining	lifelong (up to 24 months) vs. Detraining (6 months)	exercise maintained mitochondrial biogenesis (PGC-1α) and reduced SASP (IL-6, TNF-α) in the liver and perirenal fat	Sun et al.^107^
SD rats (male)	hepatic mitochondrial dysfunction	adult rats	VWR, VMR-OF, TM-END, TM-HIIT	6 weeks (voluntary vs. Treadmill-endurance vs. HIIT)	exercise (especially HIIT/Endurance) increased hepatic mitochondrial respiration and FAO; increased citrate synthase activity	Fletcher et al.^109^
Old Wistar rats	aging/DNA damage	21 months	regular treadmill running	2 months; 5 days/week; 20 m/min for 30 min	reduced 8-oxodG in nuclear and mitochondrial DNA; modulated DNA repair enzymes (OGG1, APE1, and Polβ) in the liver	Nakamoto et al.^111^
Aged SD rats	hepatic aging	20 months	medium-intensity exercise	8 weeks; treadmill running	proteomic analysis identified 26 differentially expressed proteins involved in stress response, metabolism, and redox balance	Li et al.^112^
F344 rats (male)	aging/oxidative Stress	18 months; 28 months	regular exercise training	8 weeks; treadmill running	attenuation of age-associated NF-κB activation and ROS formation; prevented IκBα degradation in the liver	Radák et al.^113^
Wistar rats (male)	NASH (high-fat diet induced)	adult rats	endurance exercise	8 weeks; treadmill; 60 min/day	altered mitochondrial phospholipidomic profile (cardiolipin levels); restored mitochondrial respiratory activity and OXPHOS	Gonçalves et al.^114^
C57BL/6N mice	PND	9 months; 18 months	prehabilitative resistance exercise (ladder-climbing)	5 weeks before abdominal surgery (laparotomy)	reduced neuroinflammation and improved mitochondrial health (membrane potential/ROS balance) and synaptic plasticity in the hippocampus	Liu et al.^123^
PolgA mutator mice	progeria/accelerated aging	started at 2 months	voluntary exercise (running wheel)	long-term (up to 7 months)	normalized the proteomic landscape in skeletal muscle and brain; prevented somatic mtDNA mutation accumulation and respiratory chain dysfunction	Ross et al.^124^
3xTg-AD mice	AD	12 months	infusion of plasma from exercised mice	10 injections (100 μL each) at 3-day intervals	increased hippocampal neuroplasticity and mitochondrial functions; suppressed GSK3β/tau and pro-apoptotic Bax/Bcl-2 pathways	Kim et al.^129^
C57BL/6 mice	brain aging/Neurogenesis	20–22 months	high-intensity exercise (HE)	3 weeks; treadmill (speed increased to 24 m/min)	failed to improve mitochondrial DNA copy number or neurogenesis; triggered pro-inflammatory NF-κB signaling in aged brains	E et al.^135^
Sprague–Dawley (SD) rats (male)	cognitive decline/aging	16–18 months	swimming exercise training	10 weeks; 60 min/day	enhanced mitochondrial quality control in the hippocampus; associated with improved lysosomal proteolysis and PGC-α levels	Luo et al.^122^
SD rats (male)	D-Galactose-induced aging	8 weeks (model initiation)	treadmill exercise + MitoQ (antioxidant)	8 weeks; exercise 30–60 min/day +100 μM MitoQ	improved memory by balancing mitochondrial dynamics (Mfn2 and Drp1) and reducing oxidative stress/neuroinflammation in the hippocampus	Jeong et al.^126^
LCR and HCR rats	AD hallmarks	18 months; 24 months	genetic selection of aerobic capacity (high vs. low)	lifelong (genetic strains)	low capacity (LCR) led to increased Aβ, tau phosphorylation, and reduced brain mitochondrial respiration/ATP production	Kugler et al.^128^
Lewis rats (female)	brain aging (cortex and cerebellum)	6 months; 15 months	long-term PA	lifelong (up to 15 months); *Ad libitum* wheel running	preservation of mitochondrial respiration; increased antioxidant capacity; balanced mitochondrial dynamics (Mfn2 and Drp1)	Mesquita et al.^133^
SD rats (male)	brain aging (hippocampus)	23 months	voluntary running (VR) vs. TM	4 weeks; RW (*ad libitum*) vs. TM (15 m/min, 20 min/day)	modulation of metabolic proteins (e.g., malate dehydrogenase, fructose-bisphosphate aldolase C) involved in energy metabolism	Kirchner et al.^134^
C57BL/6 mice	renal fibrosis/aging	18 months	incremental load training (ILT)	8 weeks; treadmill with increasing intensity	inhibition of TGF-β1/TAK1/MKK3/p38MAPK pathway; induction of autophagy (beclin1 and LC3II/I); reduced collagen deposition	Bao et al.^144^
C57BL/6 mice	renal vascular sclerosis/aging	3 months; 20 months	aerobic endurance exercise (Treadmill)	8 weeks; 5 days/week; 45–60 min/day	regulation of the PI3K/AKT/mTOR pathway; increased VEGF and JG12 expression; reduced basement membrane thickening	Bao et al.^145^
Humans (elderly adults)	chronic kidney disease (CKD)	70–89 years	structured moderate exercise	2 years; walking/aerobic (150 min/week) + strength	significant reduction in urinary biomarkers of kidney injury (e.g., NGAL, KIM-1) and inflammation; slowed eGFR decline	Sheshadri et al.^138^
Fisher 344 rats (male)	age-dependent CKD	18–21 months	treadmill exercise	12 weeks; 15–20 m/min, 15% grade, 60 min/day	no effect on age-related renal functional decline or oxidative stress; heart benefits did not extend to kidney	Moningka et al.^141^
SD rats (male)	chronic nephropathy/Aging	started at 1.5 months (lifelong)	VR vs. FR	lifelong until natural death	VR and FR were equally effective in retarding age-related glomerular sclerosis and proteinuria; improved survival	Loupal et al.^142^
SD rats (female)	renal aging/ferroptosis	8 months; 26 months	LLE vs. OMICT	LLE (18 months duration) vs. OMICT (8 months, 18 m/min)	activation of the NRF2/KEAP1/Klotho pathway; reduction of ferroptosis (ACSL4, ROS) and apoptosis; decreased SASP factors	Yuan et al.^143^
Aged rats (male)	renal fibrosis/aging	21 months	swimming exercise	12 weeks; 60 min/day; 5 days/week	activation of PPAR-α; downregulation of TGF-β1, Acta2, and Col1a; reduced lipid accumulation (TG) and oxidative stress (MDA)	Zhao et al.^146^
Humans (cohort study)	CKD development	≥18 years (adults)	regular leisure-time physical activity	15 years follow-up (cohort of 216,910 adults)	higher levels of activity were associated with lower risk of incident CKD across all ages.	Guo et al.^139^
C57BL/6 mice	cardiac mitochondrial oxidation	2 months; 25 months	habitual exercise (RW)	lifelong (23 months); *Ad libitum* wheel access	mitigated age-related protein carbonylation and nitration; preserved the OXPHOS activity and MnSOD levels	Padrão et al.^158^
C57BL/6 aged mice	cardiac senescence/dysfunction	22 months	combined exercise (endurance + resistance)	8 weeks; swimming + weighted tail running	upregulation of upstream stimulatory factor 2 (Usf2); activation of mitophagy and cardiac contraction pathways; anti-senescence effects	Han et al.^151^
ICR/CD-1 mice (male)	cardiac aging/mitochondrial decay	3 months; 20 months	endurance treadmill exercise	8 weeks; 12–15 m/min, 60 min/day, 5 days/week	regulation of p53 activity targeting SCO2; increased mitochondrial COX (cytochrome *c* oxidase) biogenesis; reduced oxidative stress	Qi et al.^153^
Fischer 344 rats	cardiac dysfunction/apoptosis	4 months; 20 months	treadmill exercise training	8 weeks; 5 days/week; 45 min/day (10 m/min for old)	improved mitochondrial function (state 3 respiration); balanced dynamics (Mfn1, Mfn2, Opa1, and Fis1); reduced Bax/Bcl-2 ratio	No et al.^154^
Wistar rats (male)	cardiac aging/mitochondrial	18 months (start) to 22 months (end)	voluntary vs. forced activity (treadmill)	4 months; voluntary (running wheel) or treadmill (continuous)	activation of the PKG-STAT3-Opa1 axis; enhancement of mitochondrial fusion and cristae integrity; reduced oxidative damage	Szekeres et al.^155^
Fischer 344 rats	cardiac apoptosis/aging	6, 16, and 24 months	observational/aging	N/A	age-related increase in cytochrome *c* release from mitochondria to cytosol; increased caspase-3 activity and DNA fragmentation	Phaneuf and Leeuwenburgh^156^
Old Wistar rats	cardiac aging/H_2_S deficiency	22–24 months	moderate exercise (treadmill)	6 weeks; 5 days/week; 15 m/min for 30 min	restoration of endogenous H_2_S synthesis (CSE and CBS levels); improved mitochondrial respiratory control ratio (RCR) and Ca^2+^ transport	Strutynska et al.^157^
SD rats (male)	cardiac aging (D-gal-induced)	8 weeks (model initiation)	treadmill exercise training	8 weeks; 60 min/day, 5 days/week (up to 20 m/min)	augmentation of Sirt1 signaling; reduction of proinflammatory cytokines (TNF-α and IL-6); inhibition of cardiac apoptosis	Chen et al.^158^
Fischer rats (male)	aging/acute oxidative stress	8 months; 24 months	acute exhaustive exercise	single bout until exhaustion (young: 25 m/min; old: 15 m/min)	aging increased mitochondrial ROS production in heart and liver; acute exercise exacerbated ROS in aged heart but not in the liver; decreased the GSH/GSSG ratio	Bejma et al.^159^
Fischer rats	cardiac aging	24 months	chronic physical exercise (treadmill)	20 min, twice daily; 5 days/week; for 12–18 months	increased succinic dehydrogenase (SDH) activity; prevention of age-related decrease in mitochondrial area and numerical density	Balietti et al.^160^
Drosophila (fruit fly)	high-salt-induced heart aging	1–7 weeks (Fly lifespan)	LTE	treadmill-like device (Tread Wheel); Daily training	prevention of CG2190 downregulation; reduced ROS and lipid peroxidation (MDA); improved cardiac contractility under salt stress	Wen et al.^161^
*Drosophila melanogaster* (fruit fly)	cardiac aging/mobility decline	young (1 week) to Old (5–7 weeks)	mechanized exercise (power tower)	2–3 h/day, 5 days/week; ramped intensity	reduced age-related decline in climbing speed and cardiac performance (systolic/diastolic diameter)	Piazza et al.^162^

RWE, Voluntary resistance wheel exercise; HIIT, High-intensity interval training; MICT, Moderate-intensity continuous training; RT, Resistance training; ET, Endurance training; LTE, Long-term exercise; ILT, Incremental load training; VWR, Voluntary wheel running; VMR-OF, Voluntary moderate running in open fieldTM-END, Treadmill endurance exercise; RW, Running wheel; TM, Treadmill; OMICT, Old moderate-intensity continuous training; VR, Voluntary running; Mfn1/2, Mitofusin 1/2; Opa1, Optic atrophy 1; Drp1, Dynamin-related protein 1; Fis1, Fission 1; LC3, Microtubule-associated protein light chain 3; Sesn2, Sestrin 2; AMPKα2, Adenosine monophosphate-activated protein kinase alpha 2 subunit; PGC-1α, Peroxisome proliferator-activated receptor gamma coactivator-1 alpha; ERRα, Estrogen-related receptor alpha; PINK1, PTEN-induced kinase 1; Parkin, Parkinson disease protein 2; p62, Sequestosome 1; SOD2, Superoxide dismutase 2; NF-κB, Nuclear factor-kappa B; ROS, Reactive oxygen species; TLR9, Toll-like receptor 9; cGAS, Cyclic GMP-AMP synthase; GDF15, Growth differentiation factor 15; mitoSCs, Mitochondrial supercomplexes; NRF-1, Nuclear respiratory factor 1; TFAM, Mitochondrial transcription factor A; OXPHOS, Oxidative phosphorylation; SIRT3, Sirtuin 3; 15-PGDH, 15-hydroxyprostaglandin dehydrogenase; VEGF, Vascular endothelial growth factor; PARKIN; Parkin RBR E3 ubiquitin protein ligase; Bmal1, Brain and muscle ARNT-like 1; IκBα, Nuclear factor of kappa light polypeptide gene enhancer in B-cells inhibitor alpha; GSK3β, Glycogen synthase kinase 3 beta; Bax/Bcl-2, Bcl-2-associated X protein/B cell lymphoma 2; TGF-β1, Transforming growth factor beta 1; TAK1, TGF-beta-activated kinase 1; MKK3, Mitogen-activated protein kinase kinase 3; p38MAPK, p38 mitogen-activated protein kinase; PI3K/AKT/mTOR, Phosphatidylinositol 3-kinase/Protein kinase B/Mammalian target of rapamycin; VEGF, Vascular endothelial growth factor; JG12, Renal juxtaglomerular cell protein 12; NGAL, Neutrophil gelatinase-associated lipocalin; KIM-1, Kidney injury molecule 1; eGFR, Estimated glomerular filtration rate; ACSL4, Acyl-CoA synthetase long-chain family member 4; NRF2/KEAP1, Nuclear factor erythroid 2-related factor 2/Kelch-like ECH-associated protein 1; Klotho, Klotho longevity protein; PPAR-α, Peroxisome proliferator-activated receptor alpha; Acta2, Actin alpha 2 smooth muscle; Col1a, Collagen type 1 alpha; TG, Triglyceride; MDA, Malondialdehyde; Usf2, Upstream stimulatory factor 2; SCO2, Synthesis of cytochrome *c* oxidase 2; PKG-STAT3, Protein kinase G/Signal transducer and activator of transcription 3; CSE/CBS, Cystathionine gamma-lyase/Cystathionine beta-synthase; RCR, Respiratory control ratio; H2S, Hydrogen sulfide; mKO, Mitochondrial knockout; KD, Knockdown; OE, Overexpression; PMM, Primary mitochondrial myopathy; mAB2, Monoclonal antibody 2; SD rats, Sprague–Dawley rats; LCR/HCR rats, Low aerobic capacity rats/High aerobic capacity rats; mtNOD, Mitochondrial non-obese diabetic; 3xTg-AD, Triple transgenic Alzheimer’s disease mice; PND, Perioperative neurocognitive disorder; MASLD, Metabolic dysfunction-associated steatotic liver disease; NASH, Non-alcoholic steatohepatitis; OVX, Ovariectomy; HFD, High-fat diet; CS, Citrate synthase; NADH-TR, NADH-tetrazolium reductase; VO2max, Maximal oxygen uptake; VO2peak, Peak oxygen uptake; 1-RM, One-repetition maximum; 31P MRS, 31Phosphorus magnetic resonance spectroscopy; PCr/ADP, Phosphocreatine/Adenosine diphosphate; PDH, Pyruvate dehydrogenase; SDH, Succinic dehydrogenase; mtDNA, Mitochondrial DNA; COX, Cytochrome *c* oxidase; TBARS, Thiobarbituric acid reactive substances; 8-oxodG, 8-oxo-2′-deoxyguanosine; OGG1, 8-oxoguanine DNA glycosylase 1; APE1, Apurinic/apyrimidinic endonuclease 1; Polβ, DNA polymerase beta; FAO, Fatty acid oxidation; CR, Caloric restriction; DR, Dietary restriction; FR, Food restriction; LLE, Life-long exercise; PA, Physical activity; SASP, Senescence-associated secretory phenotype; BMI, Body mass index; CKD, Chronic kidney disease; N/A, Not applicable; CAT, Catalase.

In other studies, targeting mitochondrial quality regulation showed a significant improvement in renal decline.^163^ Unfortunately, few studies have explained how exercise alleviates renal aging via pathways involving mitochondria, highlighting a key direction for future research. Further exploration of the precise molecular mechanisms underlying exercise-mediated renal protection may provide important insights into the prevention and management of kidney aging and related diseases. It is also worth emphasizing that exercise dosage should be carefully quantified at different stages of aging and disease progression to avoid potential renal injury.

### Exercise remodels mitochondrial quality to reverse heart aging

As the core organ responsible for lifelong blood circulation, the heart is closely associated with cardiovascular and pulmonary functions, primarily through neural regulation. With aging, declining cardiac function has become a major health concern. Statistical data indicate a global increase in the incidence of heart failure, with a marked increase in the prevalence of heart disease among the elderly population.^8^ Exercise is an effective intervention for delaying cardiac aging,^164^ and its cardioprotective effects are closely linked to the maintenance of mitochondrial quality.^160^

Experimental studies on animals have demonstrated that sedentary aged mice exhibit more pronounced myocardial damage than their active counterparts, with a substantial downregulation in mitochondrial oxidative phosphorylation activity within the myocardium.^151^ An 8-week treadmill exercise regimen in 20-month-old mice was found to reverse the decrease in cardiac mitochondrial oxygen respiration efficiency and calcium retention capacity, inhibit abnormal hydrogen peroxide accumulation, improve mitochondrial dynamics, and reduce excessive mitophagy and mitochondrial-mediated apoptosis, thereby mitigating myocardial aging. The protective effects of this intervention are likely mediated through the protein kinase G (PKG)/signal transducer and activator of transcription 3 (STAT3)/OPA1 signaling pathway, thereby optimizing mitochondrial function and enhancing ATP synthase activity (Figure 4E).^154^^,^^155^

As aging progresses, the expression of the anti-apoptotic protein Bcl-2 in the myocardial tissue of mice aged 16–24 months gradually declines, whereas the activities of Cyt-C, mitochondrial manganese superoxide dismutase, and glutathione peroxidase increase synchronously.^156^ In aged rats, exercise intervention can reverse abnormal increases in the levels of endogenous hydrogen sulfide, superoxide anion, hydrogen peroxide, diene conjugates, MDA, and mPTP opening in the myocardial mitochondria.^157^ The aerobic-resistance exercise pattern can also activate mitophagy in the myocardium of aged mice, alleviate age-induced pathological myocardial hypertrophy and oxidative stress damage, inhibit cardiac aging, and simultaneously improve overall cardiac function.^152^

An intervention study utilizing a *Drosophila* model of age-related cardiac dysfunction, developed through high salt intake and myocardial-specific overexpression of salazine, demonstrated that exercise intervention can reduce the cardiac cycle duration and decrease the arrhythmia index, MDA content, salazine expression, and dTOR levels while concurrently modulating SOD activity and PGC-1α expression. Additionally, it alters the quantitative characteristics of mitochondria and myofibrils.^161^ Furthermore, long-term exercise intervention can mitigate age-related degenerative changes in cardiac function in *Drosophila* by enhancing mitochondrial transport efficiency.^162^

A previous study using a D-galactose-induced aging mouse model showed that exercise intervention ameliorates myocardial structural disarray, enhances myocardial pathological morphology, and upregulates the expression of energy-regulating proteins, including SIRT1, PGC-1α, and 5′ AMP-activated protein kinase catalytic subunit alpha-1 (AMPKα1), thereby demonstrating a significant cardioprotective effect.^158^ Furthermore, swimming training in aged rats can restore impaired cardiac mitochondrial function by facilitating mitochondrial nitric oxide production.^165^ Collectively, regular exercise decreases the levels of ROS and MDA in the myocardium of aged mice while increasing mitochondrial membrane potential, GSH/GSSG ratio, Mn-SOD activity, mitochondrial DNA copy number, and COXII/IV expression. Additionally, it upregulates the transcriptional levels of various mitochondrial-related genes, such as PGC-1α/β, TFAM, TFB1M, and TFB2M.^153^ However, related studies indicate significant variability in these effects; notably, a previous study showed that a single acute exercise session in aged rats does not cf. a cardioprotective effect and may even aggravate myocardial tissue damage.^159^ Therefore, when using exercise to regulate mitochondrial function to delay or reverse cardiac aging, exercise intensity must be carefully considered, particularly in elderly individuals with differing exercise capacities.

## Challenges in exercise-mediated remodeling of mitochondria in high-energy-consuming tissues and organs and in delaying the aging process

Numerous studies have reported the decline in mitochondrial quality in high-energy-consuming organs due to aging, and mitochondrial quality has become a target mechanism for the anti-aging effects of exercise. The research results described in this article confirm this viewpoint. However, the application of exercise in combating aging by reshaping the quality of mitochondria in high-energy-consuming organs still faces many challenges. For example, the relationship between aging level and exercise dosage still needs to be clarified. As age increases, the ability of the body to withstand exercise intensity varies. A uniform, fixed dosage may lead to poor results or overload in people with different aging levels, which may be detrimental to the improvement of mitochondrial quality. Moreover, aging does not occur in a single high-energy-consuming organ. Therefore, it may be necessary to pay attention to the status of multiple organs during exercise for developing a plan that is conducive to high-energy-consuming organs with low thresholds. In addition, current research, particularly on the motor regulation of mitochondria in the aging heart, liver, kidneys, and brain, is mainly conducted through animal experimentation, and supporting human clinical data remain limited. Current research suggests that even in a state of low voluntary exercise motivation, forced exercise can still provide multiple benefits. However, basic research shows that it is not conducive to recovery. Therefore, enhancing voluntary exercise motivation by adjusting the exercise dosage, intervention methods, and environment during the intervention process may be beneficial to maximizing the benefits of exercise. Notably, the different genders, underlying diseases, baseline exercise level, past medical history, mental state, psychology, and personal exercise preferences of the elderly population are all important factors to consider when implementing exercise. Therefore, exercise can indeed reshape the mitochondrial quality of high-energy-consuming organs and play an important role in combating aging and preventing disease. However, from a clinical perspective, measuring the function of multiple organs and tissues may be necessary, which would require more scientific and personalized exercise prescriptions.

## Conclusion

Mitochondrial health is critical for maintaining homeostasis in eukaryotic cells, primarily through the regulation of ROS, inflammation, apoptosis, and calcium balance. Exercise is recognized as a major strategy for modulating mitochondrial quality. However, it is a non-targeted process involving multiple organs. This article summarizes the current molecular mechanisms through which exercise modulates mitochondrial quality in high-energy-consuming tissues and organs during aging. In this process, several issues warrant attention: elucidating the molecular mechanisms through which exercise influences mitochondrial quality across different organs; identifying the key mediators and signaling pathways through which exercise modulates tissue mitochondria; and exploring whether these mitochondrial mediators can serve as targets for exogenous intervention. Moreover, this study predominantly relies on animal research, with only limited evidence available from human organ systems. Notably, discrepancies exist between the findings of animal and human studies. Comprehensive health assessments must be conducted before implementing exercise interventions, particularly within the elderly population. Future efforts should focus on integrating personal medical history, blood biomarkers, and multiomics analyses to facilitate the development of precise, personalized, and targeted exercise programs.

## Acknowledgments

This project was supported by the special support plan of high-level talent introduction of 10.13039/501100009004Anhui University of Chinese Medicine (No. DT2500001553), and the young scientists research project of Anhui provincial department of education (2025AHGXZK40032). We sincerely appreciate the editors, reviewers and all behind-the-scenes staff for their diligent efforts to improve this manuscript. We thank Editage for their professional editing assistance in proofreading this manuscript.

## Author contributions

Writing – original draft, Z.Z. and J.G.; writing – review and editing, M.W. and H.Z.; funding acquisition, H.Z.; resources and supervision, M.W. and H.Z.

## Declaration of interests

The authors declare that they have no competing interests.

## Declaration of generative AI and AI-assisted technologies in the writing process

The authors declare that no artificial intelligence tools were utilized in the preparation of this manuscript.
